# A Multifunctionalized Potyvirus-Derived Nanoparticle That Targets and Internalizes into Cancer Cells

**DOI:** 10.3390/ijms25084327

**Published:** 2024-04-13

**Authors:** Daniel A. Truchado, María Juárez-Molina, Sara Rincón, Lucía Zurita, Jaime Tomé-Amat, Corina Lorz, Fernando Ponz

**Affiliations:** 1 Centro de Biotecnología y Genómica de Plantas (CBGP), Instituto Nacional de Investigación y Tecnología Agraria y Alimentaria (INIA-CSIC), Universidad Politécnica de Madrid (UPM), 28223 Pozuelo de Alarcón, Spain; daniel.truchado@upm.es (D.A.T.); mjuamol@ibmcp.upv.es (M.J.-M.); sararincre@gmail.com (S.R.); lucia.zurita@inia.csic.es (L.Z.); jaime.tome@upm.es (J.T.-A.); 2 Unidad de Innovación Biomédica, Centro de Investigaciones Energéticas, Medioambientales y Tecnológicas (CIEMAT), Avenida Complutense 40, 28040 Madrid, Spain; clorz@ciemat.es; 3 Instituto de Investigación Sanitaria Hospital 12 de Octubre (imas12), Avenida de Córdoba s/n, 28041 Madrid, Spain; 4 Centro de Investigación Biomédica en Red de Cáncer (CIBERONC), Avenida de Monforte de Lemos 3-5, 28029 Madrid, Spain

**Keywords:** turnip mosaic virus, VLP, protein A, Z domain, cetuximab, squamous cell carcinoma, viral nanoparticles

## Abstract

Plant viral nanoparticles (VNPs) are attractive to nanomedicine researchers because of their safety, ease of production, resistance, and straightforward functionalization. In this paper, we developed and successfully purified a VNP derived from turnip mosaic virus (TuMV), a well-known plant pathogen, that exhibits a high affinity for immunoglobulins G (IgG) thanks to its functionalization with the Z domain of staphylococcal Protein A via gene fusion. We selected cetuximab as a model IgG to demonstrate the versatility of this novel TuMV VNP by developing a fluorescent nanoplatform to mark tumoral cells from the Cal33 line of a tongue squamous cell carcinoma. Using confocal microscopy, we observed that fluorescent VNP–cetuximab bound selectively to Cal33 and was internalized, revealing the potential of this nanotool in cancer research.

## 1. Introduction

The use of viral nanoparticles (VNPs) in cancer research has become increasingly common in recent years owing to their biocompatibility, self-assembly, wide range of shapes, and easily modifiable compositions [1,2]. Plant VNPs are especially noteworthy as they are not infective to humans and are easily produced by molecular farming [3]. Plant-made VNPs include virions, which are infective to plants, and virus-like particles (VLPs), which have no infectious ability, as they are formed by self-assembled capsid proteins (CPs). Elongated and rod-shaped VNPs are particularly attractive for cancer research because they have shown better tumor homing abilities than other alternative shapes [4].

Turnip mosaic virus (TuMV) is one example of an elongated, flexuous plant virus whose VNPs have been used to develop a great number of nanotools with applications in different areas, including cancer research. In that case, TuMV virions that were chemically functionalized with epigallocatechin gallate (EGCG) showed good tumor homing and antiproliferative effects in Cal33 cells, which were derived from a tongue squamous cell carcinoma (SCC) [5]. In this study, we aimed to demonstrate a potential nanocarrier for antitumor drugs specifically targeting tumor cells as an example of the versatility of developing a new TuMV VLP with a high affinity for antibodies. The structure and composition of the TuMV capsid protein, composed of approximately 2000 copies of the same capsid protein, offers several readily accessible amino acids that may be amenable to conjugation with various antitumor drugs [6,7]. In addition, TuMV VNPs are longer than other flexuous viruses that have already been shown to act as good nanocarriers for antitumor drugs, such as potato virus X (PVX) and tobacco mosaic virus (TMV) [8,9]. This allows them to offer more binding sites for the drug of interest per VNP, thus reducing the effective concentration to reduce potential side effects. Moreover, as with other plant viruses, no adverse effects have been observed when TuMV VNPs were tested in mice [10,11], and their VLPs are easily induced through transient expression in *Nicotiana benthamiana* plants [7,10,12]. These features make TuMV VLPs good candidates for developing novel potential nanocarriers for antitumor drugs.

Overexpression of the epidermal growth factor receptor (EGFR) is common in SCCs, making it a good target for developing new therapeutic agents [13,14,15]. One such therapeutic agent is cetuximab, a mouse/human chimeric IgG monoclonal antibody that binds EGFR with a higher affinity than its natural ligands, transforming growth factor alpha (TGF-α) and the epidermal growth factor (EGF), and preventing them from interacting with the receptor [16,17]. It also promotes the internalization of EGFR [18]. Cetuximab has been successfully used in the treatment of SCC, as well as in other human cancers with EGFR overexpression, such as colorectal cancer [19]. As cetuximab has shown two desirable characteristics for our nanocarrier (its strong binding to EGFR and the subsequent internalization of the receptor), we chose it to functionalize TuMV VLPs. Therefore, binding cetuximab to TuMV VLPs should allow their binding to SCC cells with a high affinity and, eventually, internalize them into the cell.

In order to bind cetuximab to the surface of TuMV VLPs, the fusion of a protein sequence providing a high affinity between the antibodies and the CP was required. Protein A from *Staphylococcus aureus* is a well-known protein with five homologous immunoglobulin-binding domains (A–E) of 56–61 amino acids. These domains interact with the crystallizable fragment (Fc) of immunoglobulin G (IgG) of a wide variety of mammals without affecting their antigen-binding ability. Given that cetuximab is a mouse/human chimeric IgG, we hypothesized that Protein A would successfully attach cetuximab to the VLP through its Fc. Thus, the variable region of cetuximab would be exposed to the solvent, providing the ability to selectively bind EGFR-overexpressing tumor cells to the VLPs. Protein A has been frequently used to immobilize antibodies to a solid surface, especially for antibody purification [20]. In this study, we used the Z domain to functionalize TuMV VLPs via gene fusion and provide them with an affinity for IgGs. This Z domain is a synthetic sequence of 58 amino acids, derived from the domain B of staphylococcal Protein A, which shows more stability than the original and presents a structure of three antiparallel helixes [21,22]. Functionalization of elongated VNPs with the IgG-binding domains of Protein A is not new, and has also been performed in other plant viruses [23,24,25]. However, unlike the other plant-made VNPs, TuMV VLPs are twice as long (around 720 nm) and offer more binding sites to the IgGs, as their capsid protein is represented by approximately 2000 copies in each particle.

The functionalized TuMV VLPs–cetuximab constructs were found to interact with EGFR-overexpressing Cal33 cells derived from tongue SCCs, and their specific binding was assessed compared to VLPs conjugated to a “neutral” antibody for Cal33 cells (anti-tobacco mosaic virus; TMV) and the VLPs–Z domain constructs alone. Head and neck squamous cell carcinoma (HNSCC) is the sixth most common cancer worldwide (with 800,000 cases diagnosed and 350,000 deaths per year) [26,27]. Treatment modalities for HNSCCs mainly include surgery, radiation, chemotherapy, and epidermal growth factor receptor (EGFR)-antibody therapy in combination with cetuximab. In the last five years, immune checkpoint inhibitors (CPI) have successfully entered the second- and first-line treatment regimens for recurrent/metastatic disease. However, locoregional refractory disease and/or metastatic relapse occur in up to half of the cases, leading to a 5-year overall survival rate of 50%. Thus, new therapeutic approaches are needed to improve the poor outcomes of patients with HNSCC.

Cal33 (https://www.cellosaurus.org/CVCL_1108) (accessed 4 March 2024) is a cell line derived from a squamous cell carcinoma of the tongue [28]. This is the frequent localization of HNSCC. Cal33 is one of the best characterized HNSCC cell lines at the genomic level, including in exome, methylome, and transcriptome analyses (https://sites.broadinstitute.org/ccle/) (accessed 4 March 2024). Genomic data from this cell line can be integrated with available functional data such as drug sensitivity and knockdown/knockout data (https://depmap.org/portal/) (accessed 4 March 2024). Furthermore, Cal33 can be used in xenograft studies, where it gives rise to tumors resembling well-differentiated human HNSCC. Therefore, Cal33 is one of the most widely used HNSCC cell lines. The objective of this study was to present a novel approach to selectively functionalize TuMV VLPs by binding the antibody of interest to their surface. As a proof of concept, we also wanted to demonstrate the potential of this VLP–Z domain construct as a platform to develop a nanocarrier to target tumor cells overexpressing EGFR.

## 2. Results

### 2.1. VLP Purification and Cetuximab Functionalization Check

**Figure 1:**
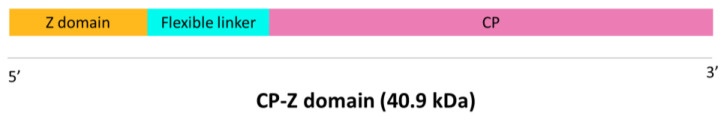
Schematic representation of the synthetic gene designed in this study. A flexible linker (GGGGSGGGGSGGGGS) was added to physically separate the two parts of the fusion protein to avoid steric hinderance. CP: TuMV capsid protein.

**Figure 2:**
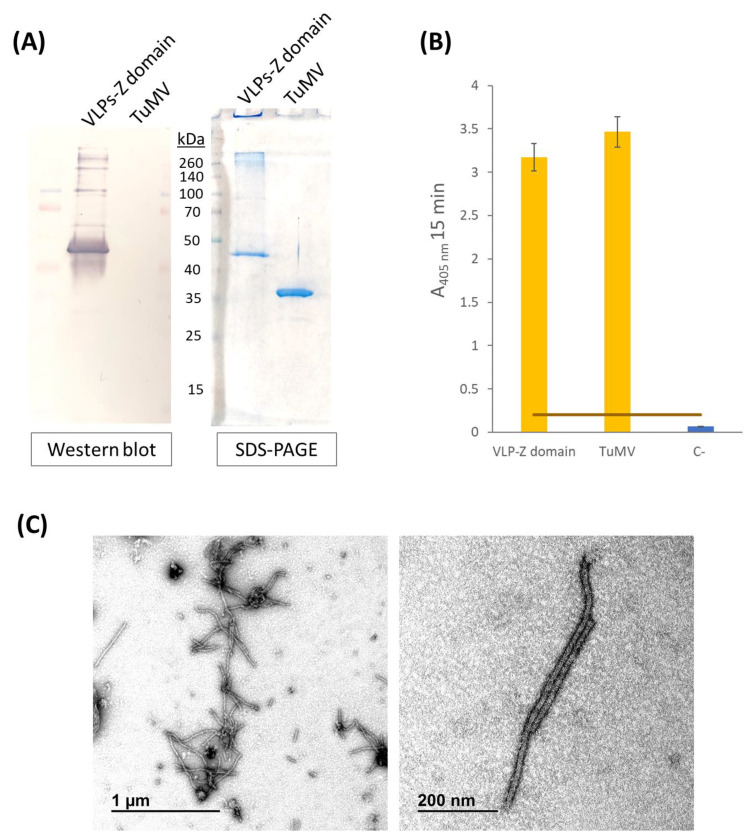
(**A**) Western blot results using anti-potyvirus as the primary antibody and stained SDS-PAGE polyacrylamide gel with the same samples. Approximate molecular weight of the different bands of the marker (Spectra^TM^ Multicolor Broad Range Protein Ladder, Fisher Scientific, Waltham, MA, USA) are highlighted. Positive control (TuMV) could not be observed in the Western blot likely due to the high affinity for IgG of the VLP-Z domain construct. (**B**) Results of the indirect ELISA carried out to chek for the presence of the CP in the purified VLPs–Z domain constructs using anti-potyvirus as the primary antibody. The error bars show the 95% confidence interval for the absorbance values. The orange bars represent the threshold for a result to be considered positive which was set at three times the value of the negative control. (**C**) Micrographs of the purified VLPs–Z domain constructs obtained by TEM. VLP: virus-like particles; TuMV: turnip mosaic virus.

The correct purification of the VLP–Z domain construct was confirmed by SDS-PAGE and Western blotting, in which a single band of the expected molecular weight for the recombinant protein CP–Z domain construct (40.9 kDa; Figure 1) was observed (Figure 2A). This result was also supported by ELISA (Figure 2B). Micrographs obtained by transmission electron microscopy (TEM) revealed the correct assembly of the VLPs (Figure 2C).

**Figure 3:**
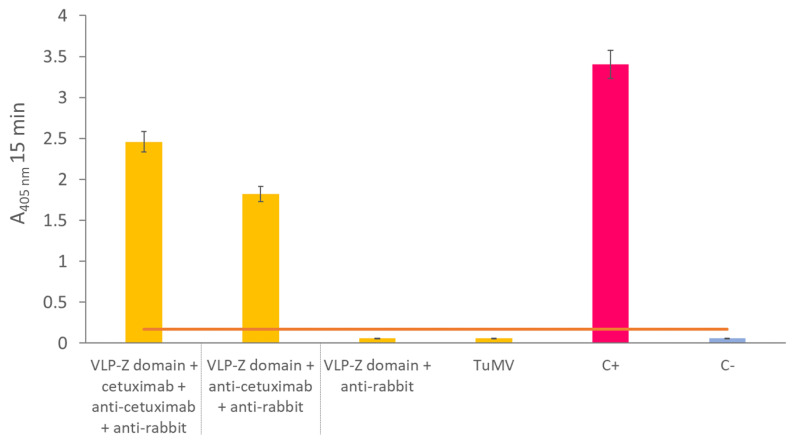
Results of the indirect ELISA in which the ability of VLPs–Z domain constructs to bind with cetuximab was tested. The first yellow column represents the result of the VLP–cetuximab complex. The error bars show the 95% confidence interval for the absorbance values. The orange bars represent the threshold for a result to be considered positive which was set at three times the value of the negative control. C+: positive control consisting of a coating step with 10 µg of free cetuximab. C-: negative control where no coating step was performed. VLP: virus-like particles; TuMV: turnip mosaic virus.

Regarding functionalization with cetuximab, the results of the indirect ELISA showed that the VLPs–Z domain constructs were able to successfully bind to cetuximab when we coated the plates with them (Figure 3).

### 2.2. Triple Functionalization of VLP-Z Domain with Cetuximab and Cy5.5

**Figure 4:**
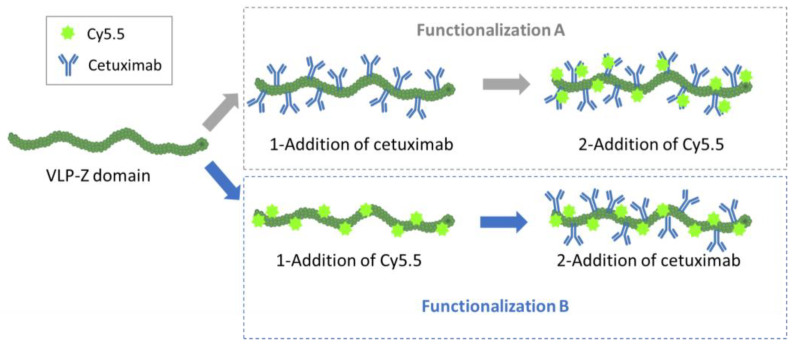
Representation of the two alternative ways through which VLP–Z domain constructs were double-functionalized with cetuximab and Cy5.5 in this study.

**Figure 5:**
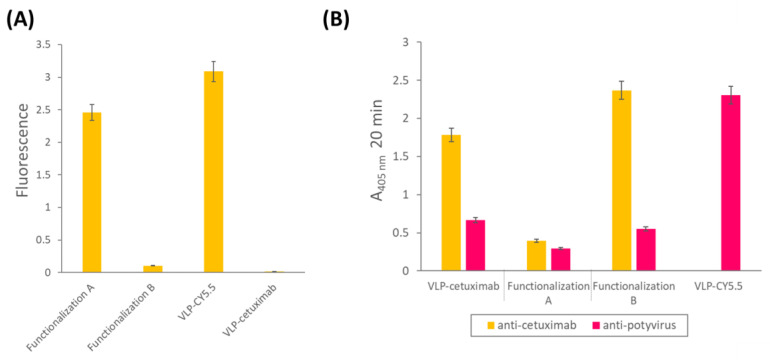
(**A**) Fluorescence values of the different functionalized VLPs obtained for the excitation and emission spectra corresponding to the Cy5.5 dye. (**B**) ELISA results using anti-cetuximab (yellow) and anti-potyvirus (pink) as primary antibodies with the different functionalized VLPs. VLP-Cy5.5 was analyzed only with anti-potyvirus. The error bars show the 95% confidence interval for the absorbance values. Functionalization A: double-functionalized VLP in which cetuximab was conjugated first and Cy5.5 afterwards. Functionalization B: double-functionalized VLP in which Cy5.5 was conjugated first and cetuximab afterwards. VLP: virus-like particle.

We observed different results depending on whether cetuximab was conjugated before (functionalization A) or after the fluorescent dye Cy5.5 (see Figure 4). The high fluorescence values of the VLP-Cy5.5 and the VLP of functionalization A (cetuximab first) showed that the fluorescent dye was correctly linked to the constructs and was detectable at the same time. However, the fluorescence detected in the case of VLP from functionalization B (Cy5.5 first) was very low indicating that conjugating cetuximab after chemically linking the Cy5.5 dye hampered the detection of the fluorescence (Figure 5A), possibly due to a quenching effect.

The indirect ELISA revealed that, despite the successful binding to the Z domain of the VLP, cetuximab could not be detected clearly when Cy5.5 was linked afterwards (functionalization A), which may be explained by the fact that the dye probably masked the epitope recognized by the primary anti-cetuximab antibody. In contrast, Cy5.5 conjugation did not hinder the detection of CP using the anti-potyvirus antibody. However, in the presence of cetuximab, the CP epitope was not detected (Figure 5B).

### 2.3. Cell Viability Assay

The results obtained from the colorimetric assay revealed that none of the constructs affected cell viability as none of them showed a significant difference to the positive control of Cal33 cells without a stimulus (Figure S1, Table S1).

### 2.4. Flow Cytometry

**Figure 6:**
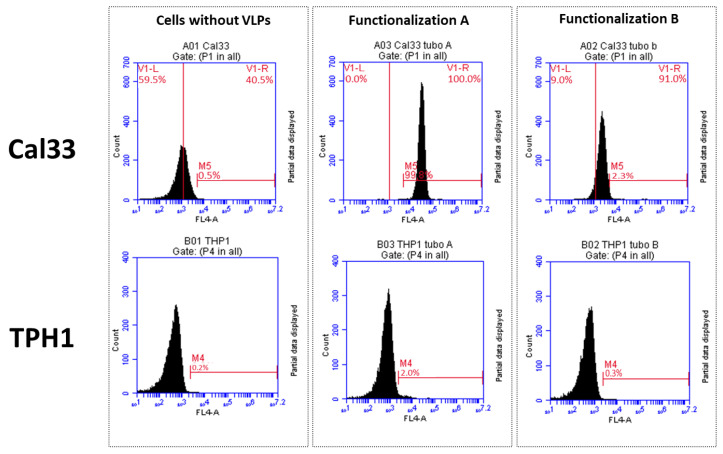
Flow cytometry analysis of VLP–cetuximab–Cy5.5 complex binding to Cal33 (EGFR+) and THP1 (EGFR-) cells. Upper row shows Cal33 cells in the absence of VLPs (**left**) and in the presence of VLPs (functionalization A, (**middle**)), (functionalization B, (**right**)). The shift in the curve observed in VLPs from functionalization A with Cal33 (upper center panel) corresponds with cells binding fluorescent protein. Functionalization A: double-functionalized VLP in which cetuximab was conjugated first and Cy5.5 afterwards. Functionalization B: double-functionalized VLP in which Cy5.5 was conjugated first and cetuximab afterwards. VLP: virus-like particle.

Results from the flow cytometry assays showed that the VLPs–cetuximab–Cy5.5 complex from functionalization A (cetuximab first) bound selectively to Cal33 cells (overexpressing EGFR) but not to THP1 (without EGFR overexpression). However, in the case of VLPs from functionalization B, the Cy5.5 dye could not be detected (Figure 6). The results for all of the negative controls were as expected (Figure S2)

### 2.5. Confocal Microscopy

**Figure 7:**
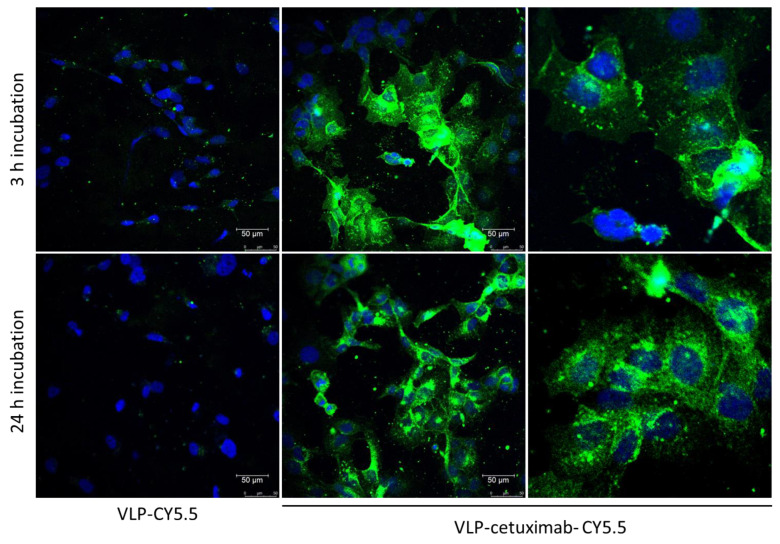
Representative images of Cal33 cells marked with fluorescent TuMV VLPs (either VLP-Cy5.5 alone or VLP-cetuximab-Cy5.5; in green) after an incubation of 3 h (**upper panels**) and 24 h (**lower panels**). The two panels on the right show enlarged images where the intracellular localization of fluorescent VLPs-cetuximab-Cy5.5 can be seen more clearly. Cell nuclei were dyed with DAPI (blue). Green areas represent the fluorescent VLPs either attached on the cell surface (cell membrane) or internalized (closer to the cell nucleus). One-micrometer-thick slices were made in the area equidistant between the apical and basal poles of the cells.

Given the results obtained with flow cytometry, we discarded the VLPs from functionalization B (Cy5.5 first) and carried out the experiment for the confocal microscopy using only VLP–cetuximab–Cy5.5 complex from functionalization A (cetuximab first). As a first experiment, we checked that free cetuximab marked with Cy5.5 bound to Cal33 cells as expected under the same conditions as the VLPs (Figure S3). Confocal microscopy images of Cal33 cells incubated with the two fluorescent VLP constructs revealed that the VLPs–cetuximab–Cy5.5 complex bound to the cells to a greater extent than VLPs–Cy5.5 (without cetuximab). Furthermore, VLPs–cetuximab–Cy5.5 appeared to enter the cells after 24 h of incubation and tended to accumulate in a perinuclear location (Figure 7). In the negative antibody control, where the VLPs–Z domain constructs were conjugated with anti-TMV (Agdia, Elkhart, IN, USA) first and Cy5.5 afterwards, binding to Cal33 was comparable to that of VLP-Cy5.5, much lower than that of VLP–cetuximab–Cy5.5 (Figure S4A,B).

## 3. Discussion

In this study, we demonstrated that TuMV VLPs can be successfully functionalized with the Z domain derived from Protein A, resulting in a highly versatile nanoplatform that can readily bind IgGs. For example, triple functionalization using cetuximab and Cy5.5 results in a fluorescent nanoparticle targeted to tumor cells with great interest in cancer research. In addition, this is the first time that functionalized TuMV VLPs have been successfully purified. So far, only TuMV VLPs without functionalization have been successfully purified following the same protocol as for virions [7] but we did not obtain the same outcome with our VLP–Z domain construct. After different modifications of the traditional purification protocol, we observed that only by extending the resuspension times of the pellets we were able to successfully purify the VLP–Z domain constructs, solving one of the major problems we faced with other TuMV VLPs [12].

The use of plant VNPs in nanomedicine has increased in recent years owing to their advantages. Plant VNP CPs self-assemble and are biosafe, biocompatible, and present a well-known structure that allows for a straightforward modification of their compositions through chemical and genetic manipulation [29,30]. One of the fields in which plant VNPs have shown great potential is in cancer research [31], where several examples of the use of plant VNPs in cancer imaging and theranostics can be found. Plant VNPs have been used in cancer research mainly as nanocarriers of drugs such as doxorubicin or cisplatin [8,32]. Doxorubicin has been the main drug used as cargo in those cases, either inside and/or outside the VNP [2]. Other antitumor compounds have also been selected for improved drug delivery using plant VNPs such as TMV and cowpea mosaic virus (CPMV) as nanocarriers, with all of them showing promising results [33,34,35,36,37,38]. Among all plant viruses, CPMV VNPs—with icosahedral capsids—stand out for the number of studies on them within cancer research, particularly in relation to their use in imaging and therapy [39]. In fact, it is the virus whose applications have gone the furthest, as it has been shown to reverse the immunosuppressive environment of tumors when used as an adjuvant in immunotherapy in canine and murine models [40]. The success of CPMV as a platform for cancer treatment is based on two intrinsic characteristics of this virus: native tropism towards vimentin on endothelial cells and their native immunostimulatory effect within solid tumors. The use of CPMV in cancer immunotherapy is an exception among icosahedral viruses, as those with high aspect ratios such as potato virus X, tobacco mosaic virus, or TuMV tend to improve drug delivery as they are retained and accumulated in tumor areas more than their icosahedral counterparts [41]. Given the elongated, flexuous capsid of TuMV together with the large amount of information available on its CP, we present TuMV-derived VLPs as a promising platform to develop alternative nanocarriers for targeted drug delivery in cancer research.

In our group, we showed that TuMV virions exhibit a good tumor-homing ability in vivo, as expected, given their elongated capsids. Moreover, the polyphenol EGCG, which has antitumor properties, was successfully bound to TuMV VNPs through chemical conjugation with lysine residues [5]. In the present study, we were able to functionalize TuMV VLPs with antibodies targeted specifically towards EGFR, which would increase their performance in a tumor environment as they will not only have a good tumor-homing ability but also a high affinity and attachment to cells overexpressing this receptor. This could be observed both in the different fluorescent marking of Cal33 when using VLPs-Cy5.5 alone or VLP–cetuximab–Cy5.5 in confocal microscopy and in the different marking of Cal33 (overexpressing EGFR) and THP1 (without EGFR) in flow cytometry. Moreover, we observed fluorescent VLPs inside Cal33 cells after 24 h of incubation using confocal microscopy, indicating that they have the potential to be used as nanocarriers and internalize drugs coating the VLP surface. TuMV VNPs stand out among other plant-made VNPs because they are the longest, offering more space to be functionalized with an antitumoral of interest. These characteristics are of great interest for cancer types where EGFR overexpression occurs, such as head and neck squamous carcinomas, glioblastomas, and lung and breast cancer [42,43,44]. This novel nanoplatform presents two main advantages for cancer research. First, we improved their tumor-homing abilities by increasing their affinity for EGFR-overexpressing cells by attaching cetuximab to their surface using the Z domain expressed on their CPs. Second, we have shown that, after binding cetuximab to the VLPs, there are still accessible residues amenable to conjugation with a compound of interest. In our case, we conjugated Cy5.5 to lysine residues, but other compounds with antitumor properties (such as EGCG) could potentially bind to VLP–cetuximab. These compounds would mainly targeted EGFR-overexpressing cells and would eventually be internalized into the cell. Because of their long capsids, of about 2000 CP copies per VLP, the novel nanotool also offers more binding sites for these antitumor compounds than other icosahedral and even flexuous viruses, thus making them good candidates for reducing the effective concentration of the compound, which is important to avoid possible side effects.

One of the main applications of the fluorescent TuMV VLP we developed is within cancer theranostics, particularly optical imaging. Although we used the fluorescence of Cy5.5 to confirm the affinity of the construct for Cal33 cells, the nanotool itself could be used to mark tumor cells with high sensitivity. Other VNPs have been used for this purpose instead of synthetic nanoparticles due to two main reasons: fewer side effects given their short circulation and retention times and their easily modifiable composition for improved targeting [2,45]. Different plant VNPs have been used for tumor imaging, with special emphasis on those derived from CPMV [39,46,47]. However, TuMV VLPs are elongated and flexuous, with all the advantages already mentioned in comparison with icosahedral viruses.

The main drawback of this study was the absence of fluorescence in the construct in which Cy5.5 was conjugated first followed by the addition of cetuximab to the VLP. This may be explained by the different sizes of cetuximab (152 kDa) compared to Cy5.5 (1.3 kDa) and their relative positions. Cetuximab would densely coat the VLP, covering the fluorescence of the Cy5.5 dye if it was conjugated first and making it undetectable in the different analyses. By conjugating Cy5.5 after cetuximab, not only did we overcome this problem but also the binding of cetuximab to EGFR was not affected.

Apart from cancer research, a nanoplatform based on VNPs with a high affinity for IgGs has multiple applications in the field of antigen sensing. Two other potyviruses (zucchini yellow mosaic virus and tobacco etch virus) functionalized with nanobodies have recently been developed through genome engineering, revealing the attractiveness of this group of plant viruses for antibody presentation [48]. In addition, our VLPs–Z domain constructs go a step further as they can readily bind other IgGs of interest and can be double-functionalized with other compounds through chemical conjugation, giving them great versatility as a novel nanoplatform.

In conclusion, we report the development and successful purification of a TuMV VLP with a high affinity for IgGs. As a proof of concept, we used cetuximab as a model IgG to develop fluorescent VLPs that specifically target cancer cells overexpressing EGFR, resulting in an interesting nanoplatform for cancer theranostics.

## 4. Materials and Methods

To develop the TuMV-based nanocarrier, we first produced and purified VLPs–Z domain constructs through transient expression in plants. Once purified, we functionalized them with cetuximab (which would provide high affinity binding for Cal33 cells) through binding the Fc region to the Z domain and with Cy5.5 (which would make them fluorescent) through chemical conjugation to the lysine residues of the CPs.

### 4.1. Cloning in Expression Vectors and Agroinfiltration

First, a synthetic gene containing the sequence of the Z domain of *Staphylococcus aureus* Protein A fused to the CP of TuMV was designed. The Z domain was fused to the N-terminus of the CP as this is the part exposed to the solvent, and both sequences were separated by a flexible linker to avoid steric hindrances (Figure 1). This synthetic gene was designed and ordered from GeneArt (Thermo Fisher Scientific, Regensburg, Germany). This gene construct was cloned in the pEAQ-HT-DEST1 vector [49] using the top 10 *Escherichia coli* cells (Thermo Fisher Scientific, Regensburg, Germany) to finally transform cells of *Agrobacterium tumefaciens* (LBA4404 strains) for transient expression in *Nicotiana benthamiana*. Transformed bacteria were stored in plates with 50 µg/mL rifampicin and 50 µg/mL kanamycin. An individual colony of transformed *A. tumefaciens* was subsequently grown for 24 h at 28 °C in LB with 50 µg/mL kanamycin up to an optical density at 600 nm (OD_600_) of 1.2. Cells were pelleted down by centrifugation and resuspended in MMA medium (10 mM MES, pH 5.6, 10 mM MgCl_2_ and 150 µM acetosyringone). The culture was then incubated for 2 h at 28 °C. Agroinfiltration in *N. benthamiana* plants took place using a needle-less syringe, following a protocol described elsewhere [12].

### 4.2. Purification of VLP-Z Domain

Agroinfiltrated leaves were harvested at 6 dpi. Then, VLP–Z domain construct was purified following a protocol described before for TuMV virions [50], with some modifications. Briefly, leaves were ground in 0.5 M potassium phosphate buffer, pH 7.5 (100 mL buffer per 50 g of leaves) using a blender. Then, the product was filtered through a masher and a gauze, and chloroform was added to the liquid phase (50 mL per 50 g of leaves). After stirring for 10 min at 4 °C, the sample was centrifuged at 500× *g* for 10 min at 4 °C to separate the different phases. Then, the aqueous upper phase was filtered through a Miracloth filter (Merck Millipore, Darmstadt, Germany) and centrifuged at 5000× *g* for 10 min at 4 °C. The supernatant was filtered through the Miracloth filter (Merck Millipore, Darmstadt, Germany) and NaCl and PEG were added to a final concentration of 4 and 6% (*w*/*v*), respectively. After stirring for 90 min at 4 °C, the sample was centrifuged at 12,000× *g* for 10 min at 4 °C. The supernatant was discarded, and the pellet was resuspended in 0.5 M potassium phosphate buffer, pH 7.5, and 0.01 M EDTA, pH 8.0, by stirring at 4 °C for 48 h. The suspension was then centrifuged at 80,000× *g* for 2 h at 4 °C. The supernatant was discarded, and the pellet was resuspended in 0.25 M potassium phosphate buffer, pH 7.5, 0.01 M EDTA by stirring at 4 °C for 5 days. Then, the suspension was subjected to an isopycnic centrifugation using CsCl at 150,000× *g* for 18 h at 4 °C in a fixed-angle rotor without a break. Finally, the band containing the VLP-Z domain was obtained by puncturing with a syringe.

### 4.3. Characterization of Purified VLPs-Z Domain

Characterization of purified VLPs was performed using SDS-PAGE, Western blot, and ELISA. Purified VLPs were subjected to SDS-PAGE in a 12% polyacrilamide gel with a stacking gel of 4% (Bio-Rad, Hercules, CA, USA). Half of the gel was transferred to a PVDF membrane (Amersham^TM^ Hybond^TM^ P 0.45 PVDF, Cytiva, Cornellà de Llobregat, Spain) to carry out a Western blot while the other half was stained with Imperial™ Protein Stain (Thermo Scientific, Waltham, MA, USA) following manufacturer’s instructions. The membrane for Western blot was blocked with 2% skimmed milk in PBS overnight. The detection of the recombinant protein CP–Z domain (40.9 kDa) was carried out by an incubation with a 1:200 dilution of the primary monoclonal antibody anti-POTY (Agdia, Elkhart, IN, USA) followed by a second incubation with a 1:500 dilution of the secondary antibody, anti-mouse, conjugated to alkaline phosphatase (Agdia, Elkhart, IN, USA). Alkaline phosphatase activity was detected using 1-Step^TM^ NBT/BCIP (Thermo Scientific, Waltham, MA, USA). Finally, we confirmed the detection of the VLPs–Z domain constructs through an indirect ELISA. We coated high binding plates (Fisher Scientific, Waltham, MA, USA) with 0.5 µg/well of VLPs–Z domain (or TuMV virions in the case of the positive control) in a final volume of 200 µL of 50 mM sodium carbonate buffer, pH 9.6. After 45 min at 37 °C, plates were washed 3 times with PBS-Tween and blocked with BSA 0.2% in 50 mM sodium carbonate buffer, pH 9.6, for 45 min at 37 °C. After the blocking step, plates were washed again with PBS-Tween 3 times. As the primary antibody, we used a 1:200 dilution of anti-POTY (Agdia, Elkhart, IN, USA) and, as the secondary antibody, we used a 1:500 dilution of anti-mouse conjugated to alkaline phosphatase (Agdia, Elkhart, IN, USA).

To confirm the correct assembly of VLPs–Z domain constructs, we observed them through transmission electron microscopy (TEM). To do so, a 400-mesh copper-carbon-coated electron microscopy grid was floated with 10 µL of the purified VLPs for 10 min. Then, the grid was washed with five drops of distilled H_2_O for 5 min each. Eventually, the grid was stained with 2% (*w*/*v*) uranyl acetate for 3 min and examined on a transmission electron microscope (JEM JEOL 1400, Tokyo, Japan) in an external service (TEM, ICTS-CNME, Madrid, Spain).

### 4.4. VLP-Z Domain Functionalization with Cetuximab

To create a nanotool based on the TuMV VLPs that binds specifically tumor cells with overexpressed EGFR, we checked whether they could bind to cetuximab through the Z domains that we fused to the CPs. To that end, we carried out an ELISA in which we coated high binding plates (Fisher Scientific, Waltham, MA, USA) with 0.5 µg/well of VLPs–Z domain. After an overnight incubation at 4 °C, plates were washed 3 times with PBS-Tween and blocked with BSA 0.2% in 50 mM sodium carbonate buffer, pH 9.6 during 1 h at 37 °C. Then, they were incubated with 10 µg/well of cetuximab for 1 h at 37 °C. After washing three times with PBS-Tween, plates were incubated with a 1:1000 dilution of the primary antibody anti-cetuximab (R&D Systems, Minneapolis, MN, USA) for 1 h at 37 °C. Finally, after other 3 washes with PBS-Tween, plates were incubated with a 1:2000 dilution of the secondary antibody anti-rabbit conjugated to alkaline phosphatase (Invitrogen, Waltham, MA, USA). In all cases, antibodies (including cetuximab) were diluted in PBS, 0.05% Tween 20, 2% PVP-40, 2 mg/mL BSA. We observed alkaline phosphatase activity using nitrophenylphosphate as a substrate and measuring the absorbance of the wells at 405 nm (SPECTROstar Nano^®^; BMG Labtech, Ortenberg, Germany). A negative control with no coating step was added. The positive control consisted of a well coated with 10 µg of free cetuximab.

As the VLPs–Z domain constructs presented high affinity for IgGs, we added two extra negative controls to this ELISA to discard false positives due to the binding of the Fc region of the primary and secondary antibodies to the Z domain that might remain free in the VLPs. For these controls, we coated with 0.5 µg/well of VLP–Z domain in a final volume of 200 µL of 50 mM sodium carbonate buffer, pH 9.6. Then, one of the negative controls was incubated with anti-cetuximab as the primary antibody (without previously adding cetuximab) and anti-rabbit conjugated to alkaline phosphatase as the secondary antibody. The other negative control was incubated only with the secondary antibody, anti-rabbit conjugated to alkaline phosphatase. Also, to rule out the idea that the affinity for IgG is not due to the TuMV CP itself, we added another negative control where we coated with 0.5 µg/well of free purified TuMV virions which were incubated with the primary and secondary antibodies to detect cetuximab.

### 4.5. Triple Functionalization of VLP-Z Domain with Cetuximab and Cy5.5

In addition to the ability of the VLPs to bind tumor cells, we wanted them to be easily traceable by confocal microscopy, so we double-functionalized them with cetuximab and the fluorescent dye Cy5.5 in a second experiment. As we did not know a priori which combination would be most efficient (cetuximab first and then Cy5.5 or the other way round), we carried out both options in parallel and called them functionalization A (cetuximab first) and B (Cy5.5 first; Figure 4). For the functionalization with the monoclonal antibody, we mixed 32 µg of purified VLP–Z domain (with and without previously linked Cy5.5) with 325 µg of cetuximab (Erbitux^®^, Merck Europe B.V., Schiphol-Rijk, The Netherlands) in a final volume of 800 µL of HEPES 10 mM. Thus, cetuximab was 50 × molar excess in relation to TuMV CP. The mixture was agitated overnight at 4 °C. The excess of cetuximab was removed by centrifuging the mixture at 100,000× *g* for 4 h at 4 °C. The pellet containing the functionalized VLPs was resuspended in 800 µL of HEPES 10 mM. On the other hand, the functionalization with the fluorescent dye took place by mixing 32 µg of purified VLP–Z domain (with and without previously linked cetuximab) with 3× molar excess of Amersham™ Cy5.5 mono NHS ester (GE Healthcare, Buckinghamshire, UK) to a final volume of 800 µL of HEPES 10 mM. This way, the fluorescent dye was able coat the surface of the VLPs through bioconjugation to lysine residues exposed to the solvent [6]. The mixture was agitated overnight at 4 °C and protected from the light. The excess of Cy5.5 was removed using centrifugal filters (Amicon ^®^ Ultra—0.5 mL Centrifugal Units 3 kDa, Merck Millipore, Darmstadt, Germany). An extra sample, with only VLP–Z domain and Cy5.5 (without cetuximab) was added as a negative control for the subsequent analysis. All samples were resuspended in a final volume of 800 µL 10 mM HEPES.

Once the VLPs were triple-functionalized, we checked for the correct conjugation of both Cy5.5 and cetuximab. For Cy5.5, fluorescence of the samples was measured (excitation = 683 nm; emission = 703 nm) using a Varioskan™ LUX multimode microplate reader (Thermo Scientific, Dreieich, Germany). In this analysis, we also included the construct with VLP–Cy5.5 alone. For cetuximab, we carried out an indirect ELISA to check for its presence in the samples. We coated high binding plates (Fisher Scientific, Waltham, MA, USA) with 0.5 µg/well of VLP–cetuximab–Cy5.5 from functionalizations A and B, and VLP–cetuximab without Cy5.5 in a final volume of 200 µL of 50 mM sodium carbonate buffer, pH 9.6. After 45 min at 37 °C, plates were washed 3 times with PBS-Tween and blocked with BSA 0.2% in 50 mM sodium carbonate buffer, pH 9.6, for 45 min at 37 °C. After the blocking step, we used a 1:1000 dilution of the primary antibody anti-cetuximab (R&D Systems, Minneapolis, MN, USA) and a 1:2000 dilution of the secondary antibody anti-rabbit conjugated to alkaline phosphatase (Invitrogen, Waltham, MA, USA). Both antibodies were diluted in PBS, 0.05% Tween 20, 2% PVP-40, 2 mg/mL BSA; incubations took place for 1 h at 37 °C and plates were washed 3 times with PBS-Tween after every incubation. We observed alkaline phosphatase activity using nitrophenylphosphate as a substrate and measuring the absorbance of the wells at 405 nm (SPECTROstar Nano^®^; BMG Labtech, Ortenberg, Germany). The same samples were analyzed in another indirect ELISA using anti-potyvirus to check for the presence of the VLP following the protocol mentioned before. In this ELISA, we also included the construct with VLP–Cy5.5 alone.

### 4.6. Cell Viability Assay

In order to rule out the idea that VLPs (either functionalized or not) are toxic for tumor cells, cell viability was determined through a colorimetric method based on the bioreduction of a tetrazolium salt (MTS) to an intensely colored formazan. Cal33 cells were plated (around 5000 cells/well) in 96-well plates. After 24 h, they were treated with decreasing concentrations of the different constructs (both VLPs–cetuximab–Cy5.5, free non-functionalized VLPs, free cetuximab and free Cy5.5), ranging from 0.01 µg/µL to 0.0012 µg/µL, in a final volume of 100 µL of Dulbecco’s Modified Eagle’s Medium (DMEM, Gibco, Thermo Fisher Scientific, Regensburg, Germany). Cells with the constructs were incubated for another 24 h. After this, 20 µL MTS Cell Titer 96^®^AQueous One Solution (Promega, Thermo Fisher Scientific, Waltham, MA, USA) was added to each well and plates were read after incubating them for 2 h at 37 °C. Each experiment was performed in triplicate.

### 4.7. Flow Cytometry

To check the specific interaction between VLPs, cetuximab, andCy5.5, we carried out a flow cytometry assay using two different cell lines: Cal33 (from squamous cell carcinoma, overexpressing EGFR) and THP1 (monocytes that do not express EGFR). Approximately 50,000 cells per experiment were incubated with 0.01 µg/mL of VLP–cetuximab–Cy5.5 (functionalizations A and B) for 40 min at RT. We used a BD Accuri™ C6 Plus Personal Flow Cytometer (BD Biosciences, Franklin Lakes, NJ, USA). We used near-red laser light as background noise and far-red laser to check for the presence of Cy5.5.

### 4.8. Confocal Microscopy

Cal33 cells were seeded on round glass covers in a 24-well plate (approximately 300,000 cells/well) in Dulbecco’s Modified Eagle’s Medium (DMEM, Gibco, Thermo Fisher Scientific, Regensburg, Germany) and were incubated for 24 h at 37 °C. In half of the wells, 1 µg of each construct was added, and cells were incubated for 3 h at 37 °C. After this, the medium with the construct was aspirated, cells were washed, and the medium was changed to new Dulbecco’s Modified Eagle’s Medium (DMEM, Gibco, Thermo Fisher Scientific, Regensburg, Germany). Cells were incubated for another 24 h before preparing them for microscopy. In the other half of the wells, the medium was aspirated and changed after 24 h, and 1 µg of each construct was added 3 h before preparing them for microscopy, during which they were incubated at 37 °C.

As a negative antibody control, we added a new construct for confocal microscopy in which we conjugated 32 µg of VLP-Z domain with 1 µg of anti-TMV (Agdia, Elkhart, IN, USA) and Cy5.5 following the same protocol as functionalization A with cetuximab. This construct (VLP–anti-TMV) was analyzed with an indirect ELISA using the secondary anti-rabbit conjugated to alkaline phosphatase to check for the correct binding of anti-TMV (Figure S4C). Then, Cal33 cells were incubated for 3 h and 24 h with VLP–anti-TMV following the aforementioned protocol with the rest of the constructs.

All Cal33 cells were washed 3 times with PBS and stained with DAPI. After washing 3 times with PBS, cells were fixed with 4% paraformaldehyde and glass covers were mounted with ProLong™ Gold (Thermo Fisher Scientific, Waltham, MA, USA) following manufacturer’s instructions. Micrographs were obtained using a fluorescence confocal microscope Leica TSC-SP8 (Leica, Wetzlar, Germany).

Images were obtained with a Leica TCS-SP8 confocal microscope using 405, 488, 561, and 633 nm laser excitations. All images were taken at the medium points between the apical and basal sides of the tissue using a pinhole gap of 1 µm. Graphical material was analyzed using Leica LAS X version 5.1.0 software. At least 10 images were taken for each sample and one representative image from each construct and incubation time is shown in Figure 6.

## Data Availability

Data is contained within the article and Supplementary Material.

## Supplementary Materials

The following supporting information can be downloaded at: https://www.mdpi.com/article/10.3390/ijms25084327/s1.
