# Supplementary material for: A Multifunctionalized Potyvirus-Derived Nanoparticle That Targets and Internalizes into Cancer Cells
Source: Int J Mol Sci. 2024 Apr 13;25(8):4327. doi: 10.3390/ijms25084327 (PMC11050569; doi:10.3390/ijms25084327)
Supplement: Supplementary file 1 [file ijms-25-04327-s001.zip › ijms-2883858-supplementary.pdf]

Supplementary material

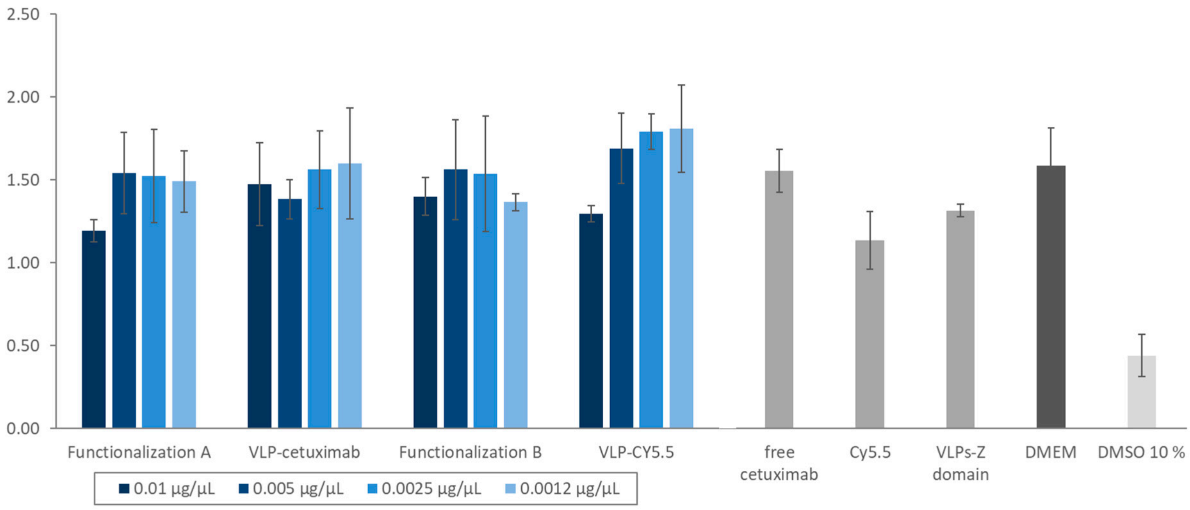

Figure S1. Absorbance values obtained from the cell viability assay using Cal 33 cells incubated with escalating concentrations of the different constructs created in this study (blue). The different controls are shown in gray. The error bars show the standard deviation of the triplicates for each experiment. DMEM: Cal 33 cells without any construct; DMSO 10 % Cal 33 cells incubated with DMSO 10 %.

Table S1. Results of a one-tailed Student's t-test analysis, assuming independent samples with unequal variance, comparing the absorbance values obtained from a cell viability analysis (MTS) of cells from the line Cal 33 exposed to the different constructs created in this study. C-: cells without any construct; C+: cells incubated with DMSO 10 %.

	**Concentration**	**C- (p-value)**	**C+ (p-value)**
Functionalization A	0.01 μg/μL	0.001	0.047
	0.005 μg/μL	0.003	0.466
	0.0025 μg/μL	0.005	0.440
	0.0012 μg/μL	0.001	0.353
VLP-cetuximab	0.01 μg/μL	0.004	0.345
	0.005 μg/μL	0.000	0.157
	0.0025 μg/μL	0.002	0.490
	0.0012 μg/μL	0.008	0.434
Functionalization B	0.01 μg/μL	0.000	0.175
	0.005 μg/μL	0.006	0.495
	0.0025 μg/μL	0.011	0.465
	0.0012 μg/μL	0.001	0.134
VLP-CY5.5	0.01 μg/μL	0.001	0.086

0.005 µg/µL	0.001	0.249
0.0025 µg/µL	0.000	0.103
0.0012 µg/µL	0.002	0.139

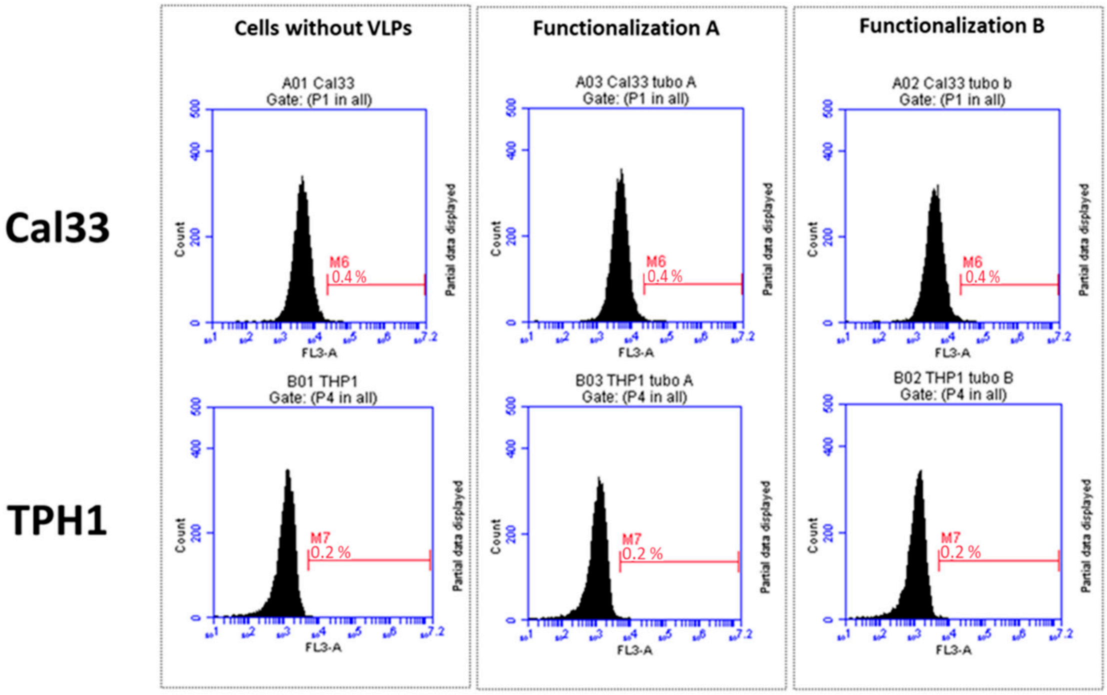

Figure S2. Negative controls from flow cytometry analysis of VLP-*cetuximab*-Cy5.5 binding to Cal33 (EFGR+) and THP1 (EGFR-) cells. Upper row shows Cal33 cells in the absence of VLPs (left) and in the presence of VLPs (Functi*onalization* A, middle), (Functional*ization* B, right). Lower row shows equivalent samples in THP1 cells.

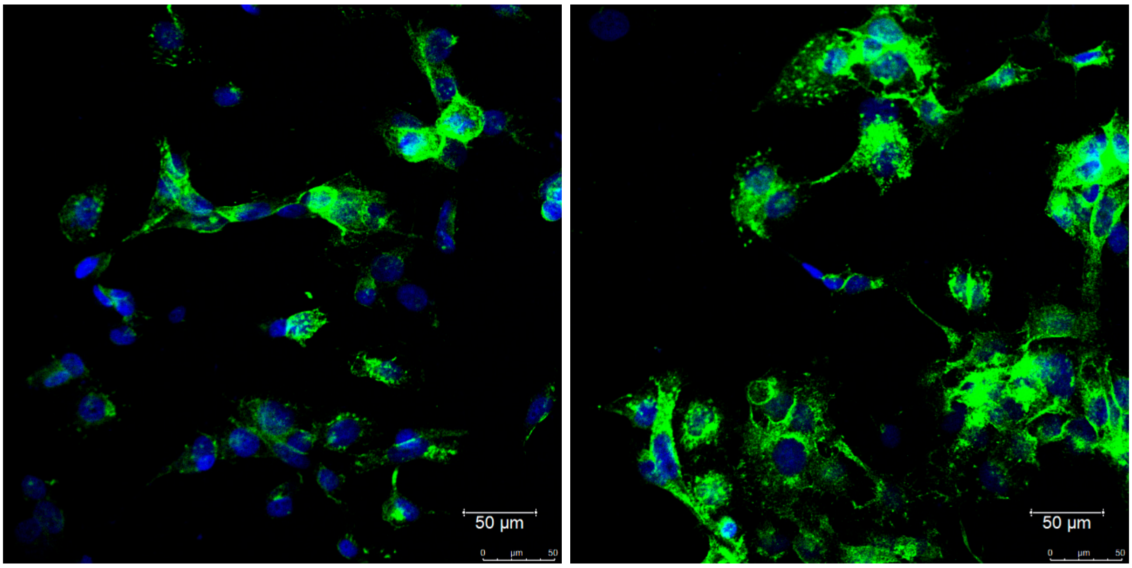

Figure S3. Cal33 cells marked with fluorescent free cetuximab-CY5.5 (green) after an incubation of 3 h (left panel) and 24 h (right panel). Cell nuclei were dyed with DAPI (blue)
